# {4-Bromo-2-[(5-chloro-2-oxidophen­yl)imino­methyl]­phenolato-κ^3^ *O*,*N*,*O*′}(methanol-κ*O*)(methano­lato-κ*O*)­oxidovanadium(V)

**DOI:** 10.1107/S160053681200311X

**Published:** 2012-01-31

**Authors:** Gholam Hossein Shahverdizadeh, Seik Weng Ng, Edward R. T. Tiekink, Babak Mirtamizdoust

**Affiliations:** a Department of Chemistry, Faculty of Science, Tabriz Branch, Islamic Azad University, PO Box 1655, Tabriz, Iran; b Department of Chemistry, University of Malaya, 50603 Kuala Lumpur, Malaysia; c Chemistry Department, Faculty of Science, King Abdulaziz University, PO Box 80203 Jeddah, Saudi Arabia; d Department of Inorganic Chemistry, Faculty of Chemistry, University of Tabriz, PO Box 5166616471, Tabriz, Iran

## Abstract

The title Schiff base complex, [V(C_13_H_7_BrClNO_2_)(CH_3_O)O(CH_3_OH)], features a vanadyl group, a tridentate Schiff base ligand, and coordinated methanol and methano­late ligands. The NO_5_ donor set is based on a distorted octa­hedron. Helical supra­molecular chains along [010] are found in the crystal structure mediated by O—H⋯O hydrogen bonds formed between the coordinating methanol mol­ecule and the phenolate O atom of the chloro­benzene residue.

## Related literature

For the structures of (*E*)-2-(2-hy­droxy­benzyl­idene­amino)­phenolates containing halide atoms on the aromatic ring(s), see: Yenişehirli *et al.* (2010 ▶). For related Schiff base vanadyl complexes containing alcohol and alkoxide ligands, see: Hartung *et al.* (2007 ▶); Clague *et al.* (1993 ▶). For the crystallization procedure, see: Harrowfield *et al.* (1996 ▶).
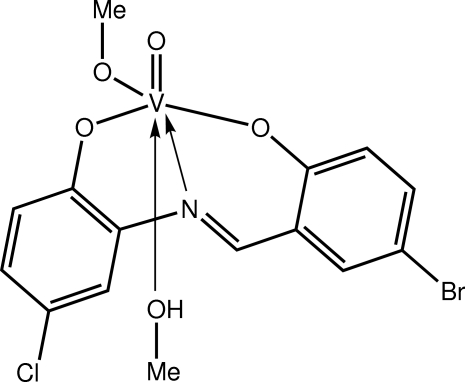


## Experimental

### 

#### Crystal data

[V(C_13_H_7_BrClNO_2_)(CH_3_O)O(CH_4_O)]
*M* *_r_* = 454.57
Monoclinic, 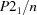

*a* = 9.9585 (2) Å
*b* = 9.8949 (2) Å
*c* = 17.3612 (3) Å
β = 100.746 (2)°
*V* = 1680.74 (6) Å^3^
*Z* = 4
Cu *K*α radiation
μ = 9.42 mm^−1^
*T* = 100 K
0.20 × 0.20 × 0.02 mm

#### Data collection

Agilent SuperNova Dual diffractometer with an Atlas detector
Absorption correction: multi-scan (*CrysAlis PRO*; Agilent, 2010 ▶) *T*_min_ = 0.255, *T*_max_ = 0.834
6974 measured reflections
3453 independent reflections
3125 reflections with *I* > 2σ(*I*)
*R*_int_ = 0.029

#### Refinement

*R*[*F* ^2^ > 2σ(*F* ^2^)] = 0.046
*wR*(*F* ^2^) = 0.131
*S* = 1.04
3453 reflections
221 parameters
1 restraint
H atoms treated by a mixture of independent and constrained refinement
Δρ_max_ = 1.37 e Å^−3^
Δρ_min_ = −0.93 e Å^−3^

### 

Data collection: *CrysAlis PRO* (Agilent, 2010 ▶); cell refinement: *CrysAlis PRO*; data reduction: *CrysAlis PRO*; program(s) used to solve structure: *SHELXS97* (Sheldrick, 2008 ▶); program(s) used to refine structure: *SHELXL97* (Sheldrick, 2008 ▶); molecular graphics: *ORTEP-3* (Farrugia, 1997 ▶) and *DIAMOND* (Brandenburg, 2006 ▶); software used to prepare material for publication: *publCIF* (Westrip, 2010 ▶).

## Supplementary Material

Crystal structure: contains datablock(s) global, I. DOI: 10.1107/S160053681200311X/hg5167sup1.cif

Structure factors: contains datablock(s) I. DOI: 10.1107/S160053681200311X/hg5167Isup2.hkl

Additional supplementary materials:  crystallographic information; 3D view; checkCIF report

## Figures and Tables

**Table 1:** Selected bond lengths (Å)

V—O1	1.872 (2)
V—O2	1.937 (2)
V—O3	2.266 (2)
V—O4	1.766 (2)
V—O5	1.596 (2)
V—N1	2.170 (3)

**Table 2:** Hydrogen-bond geometry (Å, °)

*D*—H⋯*A*	*D*—H	H⋯*A*	*D*⋯*A*	*D*—H⋯*A*
O3—H3⋯O2^i^	0.84 (1)	1.89 (2)	2.702 (3)	163 (5)

Symmetry code: (i) 
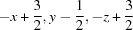
.

**Fig. 1.**
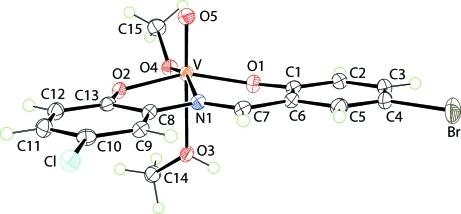
The molecular of (I) showing the atom-labelling scheme and displacement ellipsoids at the 50% probability level.

**Fig. 2.**
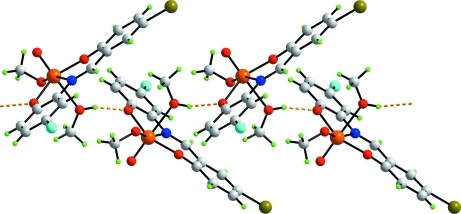
A view of the helical supramolecular chain along [010] in (I). The O—H···O hydrogen bonds are shown as orange dashed lines.

## supplementary crystallographic information

### Comment

Structural studies of complexes with (*E*)-2-(2-hydroxybenzylideneamino)phenolates containing halide atoms on the aromatic ring(*s*) are comparatively rare (Yenişehirli *et al.*, 2010) and those of complexes containing the (*E*)-4-bromo-2-((5-chloro-2-hydroxyphenylimino)methyl)phenolate ligand have not been reported. Herein we report the oxo-vanadium(V) complex of this ligand, (I).

The V atom in (I) is coordinated by the *O*,*N*,*O*-tridentate Schiff base ligand, an oxido-O atom, an O atom of the methoxido ligand and an O atom of the methanol ligand. The resulting NO_5_ donor set is based on an octahedron. The methanol ligand is *trans* to the oxido group and the *V*—*O*(methanol) bond length is significantly longer than the *V*—*O*(methanolate) bond, Table 1. The coordination geometry resembles those found in related V═O Schiff base compounds containing neutral and anionic forms of alcohols (Clague *et al.*, 1993; Hartung *et al.*, 2007).

The most prominent feature of the crystal packing is the formation of helical supramolecular chains along [010], Fig. 1 and Table 2. The connections between molecules are of the type O—H···O and involve the coordinated methanol molecule as the donor and the phenoxide-O atom of the chloro-substituted benzene ring as the acceptor.

### Experimental

A solution of 4-chlorosalicylaldehyde (10 mmol) in EtOH (25 ml) was added drop-wise to a solution of 2-(aminomethyl)-4-bromophenol (10 mmol) in EtOH (15 ml). The mixture was refluxed for 5 h. The yellow precipitate was removed by filtration and recrystallized from MeOH solution. Then the ligand (0.8 mmol) was placed in one arm of a branched tube (Harrowfield *et al.*, 1996) and oxovanadium(IV) bis(acetylacetonate) (0.8 mmol) placed in the other. Methanol was then added to fill both arms. The tube was sealed and the ligand-containing arm immersed in a bath at 333 K, while the other was left at ambient temperature. After two weeks, crystals were deposited in the arm held at ambient temperature. They were filtered off, washed with acetone and ether, and air-dried. Yield: 61%. *M*.pt.: 517 K.

### Refinement

Carbon-bound H-atoms were placed in calculated positions [C—H 0.95–0.98 Å, *U*_iso_(H) 1.2–1.5*U*_eq_(C)] and were included in the refinement in the riding model approximation. The hydroxy H-atom was located in a difference Fourier map and was refined with a distance restraint of O–H 0.84±0.01 Å; *U*_iso_ was refined. The final difference Fourier map had a peak *ca* 1 Å from Br.

### Crystal data

**Table:**

[V(C_13_H_7_BrClNO_2_)(CH_3_O)O(CH_4_O)]	*F*(000) = 904
*M**_r_* = 454.57	*D*_x_ = 1.796 Mg m^−^^3^
Monoclinic, *P*2_1_/*n*	Cu *K*α radiation, λ = 1.54184 Å
Hall symbol: -P 2yn	Cell parameters from 3765 reflections
*a* = 9.9585 (2) Å	θ = 4.5–76.3°
*b* = 9.8949 (2) Å	µ = 9.42 mm^−^^1^
*c* = 17.3612 (3) Å	*T* = 100 K
β = 100.746 (2)°	Plate, brown
*V* = 1680.74 (6) Å^3^	0.20 × 0.20 × 0.02 mm
*Z* = 4	

### Data collection

**Table:**

Agilent SuperNova Dual diffractometer with an Atlas detector	3453 independent reflections
Radiation source: SuperNova (Cu) X-ray Source	3125 reflections with *I* > 2σ(*I*)
Mirror	*R*_int_ = 0.029
Detector resolution: 10.4041 pixels mm^-1^	θ_max_ = 76.5°, θ_min_ = 4.8°
ω scan	*h* = −12→12
Absorption correction: multi-scan (CrysAlis PRO; Agilent, 2010)	*k* = −12→12
*T*_min_ = 0.255, *T*_max_ = 0.834	*l* = −10→21
6974 measured reflections	

### Refinement

**Table:**

Refinement on *F*^2^	Primary atom site location: structure-invariant direct methods
Least-squares matrix: full	Secondary atom site location: difference Fourier map
*R*[*F*^2^ > 2σ(*F*^2^)] = 0.046	Hydrogen site location: inferred from neighbouring sites
*wR*(*F*^2^) = 0.131	H atoms treated by a mixture of independent and constrained refinement
*S* = 1.04	*w* = 1/[σ^2^(*F*_o_^2^) + (0.0869*P*)^2^ + 1.3682*P*] where *P* = (*F*_o_^2^ + 2*F*_c_^2^)/3
3453 reflections	(Δ/σ)_max_ = 0.001
221 parameters	Δρ_max_ = 1.37 e Å^−^^3^
1 restraint	Δρ_min_ = −0.93 e Å^−^^3^

### Fractional atomic coordinates and isotropic or equivalent isotropic displacement parameters (Å^2^)

**Table:**

	*x*	*y*	*z*	*U*_iso_*/*U*_eq_	
Br	0.21340 (4)	0.40275 (4)	0.54299 (2)	0.03558 (16)	
V	0.58605 (5)	1.02259 (5)	0.71783 (3)	0.01590 (16)	
Cl	0.86189 (8)	1.03418 (8)	0.37755 (4)	0.02228 (19)	
O1	0.4952 (2)	0.8625 (2)	0.73417 (12)	0.0202 (4)	
O2	0.7315 (2)	1.1284 (2)	0.68762 (12)	0.0177 (4)	
O3	0.7673 (2)	0.8869 (2)	0.76214 (13)	0.0194 (4)	
H3	0.752 (5)	0.8057 (17)	0.771 (3)	0.041 (13)*	
O4	0.6177 (2)	1.0892 (2)	0.81386 (13)	0.0211 (5)	
O5	0.4594 (2)	1.1120 (2)	0.67670 (13)	0.0220 (5)	
N1	0.6091 (3)	0.9280 (3)	0.60826 (15)	0.0169 (5)	
C1	0.4304 (3)	0.7660 (3)	0.68900 (17)	0.0181 (6)	
C2	0.3401 (3)	0.6813 (3)	0.72029 (19)	0.0216 (6)	
H2	0.3230	0.6980	0.7715	0.026*	
C3	0.2760 (3)	0.5739 (3)	0.6771 (2)	0.0234 (7)	
H3A	0.2160	0.5164	0.6986	0.028*	
C4	0.3008 (3)	0.5516 (3)	0.6017 (2)	0.0237 (7)	
C5	0.3868 (3)	0.6329 (3)	0.56906 (19)	0.0218 (6)	
H5	0.4016	0.6157	0.5175	0.026*	
C6	0.4529 (3)	0.7419 (3)	0.61230 (18)	0.0191 (6)	
C7	0.5457 (3)	0.8232 (3)	0.57644 (17)	0.0182 (6)	
H7	0.5606	0.7972	0.5260	0.022*	
C8	0.7007 (3)	1.0031 (3)	0.57077 (18)	0.0171 (6)	
C9	0.7317 (3)	0.9777 (3)	0.49641 (17)	0.0172 (6)	
H9	0.6910	0.9041	0.4654	0.021*	
C10	0.8226 (3)	1.0625 (3)	0.46973 (17)	0.0189 (6)	
C11	0.8838 (3)	1.1709 (3)	0.51455 (18)	0.0212 (6)	
H11	0.9462	1.2277	0.4946	0.025*	
C12	0.8540 (3)	1.1961 (3)	0.58772 (18)	0.0202 (6)	
H12	0.8950	1.2701	0.6182	0.024*	
C13	0.7623 (3)	1.1110 (3)	0.61637 (17)	0.0169 (6)	
C14	0.8841 (3)	0.9337 (4)	0.8166 (2)	0.0259 (7)	
H14A	0.9514	0.8607	0.8278	0.039*	
H14B	0.8559	0.9619	0.8653	0.039*	
H14C	0.9250	1.0107	0.7939	0.039*	
C15	0.5543 (4)	1.2004 (4)	0.84499 (19)	0.0256 (7)	
H15A	0.5968	1.2136	0.9001	0.038*	
H15B	0.4567	1.1817	0.8412	0.038*	
H15C	0.5660	1.2822	0.8152	0.038*	

### Atomic displacement parameters (Å^2^)

**Table:**

	*U*^11^	*U*^22^	*U*^33^	*U*^12^	*U*^13^	*U*^23^
Br	0.0332 (2)	0.0270 (2)	0.0455 (3)	−0.01347 (15)	0.00452 (18)	−0.01039 (15)
V	0.0176 (3)	0.0136 (3)	0.0168 (3)	−0.00055 (19)	0.0040 (2)	−0.00131 (18)
Cl	0.0255 (4)	0.0234 (4)	0.0193 (4)	0.0001 (3)	0.0078 (3)	0.0003 (3)
O1	0.0231 (11)	0.0182 (10)	0.0201 (10)	−0.0041 (9)	0.0057 (8)	−0.0012 (8)
O2	0.0203 (10)	0.0146 (10)	0.0188 (10)	−0.0018 (8)	0.0051 (8)	−0.0001 (8)
O3	0.0208 (11)	0.0135 (10)	0.0228 (10)	−0.0017 (8)	0.0008 (8)	0.0025 (8)
O4	0.0231 (11)	0.0203 (11)	0.0209 (10)	−0.0001 (9)	0.0068 (8)	−0.0028 (8)
O5	0.0212 (11)	0.0203 (11)	0.0241 (11)	0.0011 (9)	0.0035 (9)	−0.0008 (8)
N1	0.0175 (12)	0.0151 (12)	0.0182 (11)	0.0005 (10)	0.0036 (9)	−0.0008 (9)
C1	0.0169 (13)	0.0140 (14)	0.0225 (14)	0.0021 (11)	0.0017 (11)	0.0013 (11)
C2	0.0185 (14)	0.0193 (15)	0.0276 (15)	−0.0004 (12)	0.0058 (12)	0.0011 (12)
C3	0.0174 (15)	0.0196 (15)	0.0334 (17)	−0.0030 (12)	0.0052 (12)	0.0026 (13)
C4	0.0184 (15)	0.0161 (14)	0.0338 (17)	−0.0032 (12)	−0.0025 (12)	−0.0014 (13)
C5	0.0213 (15)	0.0183 (15)	0.0244 (15)	0.0007 (12)	0.0006 (12)	−0.0023 (12)
C6	0.0179 (14)	0.0146 (14)	0.0242 (14)	−0.0001 (12)	0.0024 (11)	0.0018 (11)
C7	0.0198 (14)	0.0160 (14)	0.0190 (13)	0.0020 (12)	0.0039 (11)	0.0003 (11)
C8	0.0178 (14)	0.0132 (13)	0.0203 (14)	0.0002 (11)	0.0038 (11)	0.0005 (11)
C9	0.0203 (15)	0.0142 (14)	0.0171 (14)	0.0016 (11)	0.0031 (11)	−0.0001 (10)
C10	0.0197 (14)	0.0201 (15)	0.0167 (13)	0.0032 (12)	0.0031 (11)	−0.0003 (11)
C11	0.0220 (15)	0.0202 (15)	0.0219 (14)	−0.0014 (12)	0.0055 (11)	0.0040 (12)
C12	0.0205 (14)	0.0169 (14)	0.0224 (14)	−0.0014 (12)	0.0016 (11)	−0.0012 (11)
C13	0.0173 (14)	0.0148 (13)	0.0181 (13)	0.0023 (11)	0.0019 (11)	0.0012 (11)
C14	0.0204 (16)	0.0208 (15)	0.0322 (17)	−0.0014 (13)	−0.0063 (13)	0.0010 (13)
C15	0.0266 (16)	0.0259 (16)	0.0260 (15)	0.0014 (14)	0.0092 (13)	−0.0092 (13)

### Geometric parameters (Å, °)

**Table:**

Br—C4	1.906 (3)	C4—C5	1.372 (5)
V—O1	1.872 (2)	C5—C6	1.405 (4)
V—O2	1.937 (2)	C5—H5	0.9500
V—O3	2.266 (2)	C6—C7	1.451 (4)
V—O4	1.766 (2)	C7—H7	0.9500
V—O5	1.596 (2)	C8—C13	1.400 (4)
V—N1	2.170 (3)	C8—C9	1.405 (4)
Cl—C10	1.740 (3)	C9—C10	1.377 (4)
O1—C1	1.324 (4)	C9—H9	0.9500
O2—C13	1.340 (4)	C10—C11	1.397 (4)
O3—C14	1.433 (4)	C11—C12	1.380 (4)
O3—H3	0.835 (10)	C11—H11	0.9500
O4—C15	1.424 (4)	C12—C13	1.400 (4)
N1—C7	1.284 (4)	C12—H12	0.9500
N1—C8	1.425 (4)	C14—H14A	0.9800
C1—C6	1.411 (4)	C14—H14B	0.9800
C1—C2	1.411 (4)	C14—H14C	0.9800
C2—C3	1.385 (5)	C15—H15A	0.9800
C2—H2	0.9500	C15—H15B	0.9800
C3—C4	1.396 (5)	C15—H15C	0.9800
C3—H3A	0.9500		
			
O5—V—O4	101.68 (11)	C6—C5—H5	120.1
O5—V—O1	99.96 (11)	C5—C6—C1	119.6 (3)
O4—V—O1	100.33 (10)	C5—C6—C7	118.0 (3)
O5—V—O2	98.49 (11)	C1—C6—C7	122.5 (3)
O4—V—O2	92.46 (10)	N1—C7—C6	124.4 (3)
O1—V—O2	154.86 (10)	N1—C7—H7	117.8
O5—V—N1	92.90 (11)	C6—C7—H7	117.8
O4—V—N1	163.59 (11)	C13—C8—C9	120.5 (3)
O1—V—N1	84.30 (10)	C13—C8—N1	112.9 (3)
O2—V—N1	77.84 (9)	C9—C8—N1	126.5 (3)
O5—V—O3	173.36 (10)	C10—C9—C8	118.1 (3)
O4—V—O3	84.85 (10)	C10—C9—H9	120.9
O1—V—O3	79.86 (9)	C8—C9—H9	120.9
O2—V—O3	79.85 (9)	C9—C10—C11	121.8 (3)
N1—V—O3	80.47 (9)	C9—C10—Cl	119.1 (2)
C1—O1—V	135.74 (19)	C11—C10—Cl	119.1 (2)
C13—O2—V	119.48 (19)	C12—C11—C10	120.3 (3)
C14—O3—V	122.00 (19)	C12—C11—H11	119.8
C14—O3—H3	110 (3)	C10—C11—H11	119.8
V—O3—H3	118 (4)	C11—C12—C13	119.0 (3)
C15—O4—V	129.3 (2)	C11—C12—H12	120.5
C7—N1—C8	122.0 (3)	C13—C12—H12	120.5
C7—N1—V	127.0 (2)	O2—C13—C12	121.8 (3)
C8—N1—V	110.89 (19)	O2—C13—C8	117.9 (3)
O1—C1—C6	122.4 (3)	C12—C13—C8	120.3 (3)
O1—C1—C2	118.4 (3)	O3—C14—H14A	109.5
C6—C1—C2	119.2 (3)	O3—C14—H14B	109.5
C3—C2—C1	120.7 (3)	H14A—C14—H14B	109.5
C3—C2—H2	119.7	O3—C14—H14C	109.5
C1—C2—H2	119.7	H14A—C14—H14C	109.5
C2—C3—C4	119.0 (3)	H14B—C14—H14C	109.5
C2—C3—H3A	120.5	O4—C15—H15A	109.5
C4—C3—H3A	120.5	O4—C15—H15B	109.5
C5—C4—C3	121.8 (3)	H15A—C15—H15B	109.5
C5—C4—Br	119.4 (3)	O4—C15—H15C	109.5
C3—C4—Br	118.8 (3)	H15A—C15—H15C	109.5
C4—C5—C6	119.7 (3)	H15B—C15—H15C	109.5
C4—C5—H5	120.1		
			
O5—V—O1—C1	69.0 (3)	C2—C3—C4—C5	0.0 (5)
O4—V—O1—C1	172.9 (3)	C2—C3—C4—Br	179.5 (2)
O2—V—O1—C1	−67.7 (4)	C3—C4—C5—C6	0.2 (5)
N1—V—O1—C1	−23.0 (3)	Br—C4—C5—C6	−179.2 (2)
O3—V—O1—C1	−104.3 (3)	C4—C5—C6—C1	0.3 (5)
O5—V—O2—C13	−82.2 (2)	C4—C5—C6—C7	178.4 (3)
O4—V—O2—C13	175.6 (2)	O1—C1—C6—C5	176.3 (3)
O1—V—O2—C13	54.6 (3)	C2—C1—C6—C5	−1.0 (4)
N1—V—O2—C13	8.9 (2)	O1—C1—C6—C7	−1.7 (5)
O3—V—O2—C13	91.2 (2)	C2—C1—C6—C7	−179.1 (3)
O4—V—O3—C14	−36.8 (2)	C8—N1—C7—C6	179.1 (3)
O1—V—O3—C14	−138.3 (2)	V—N1—C7—C6	−5.0 (4)
O2—V—O3—C14	56.6 (2)	C5—C6—C7—N1	177.6 (3)
N1—V—O3—C14	135.9 (2)	C1—C6—C7—N1	−4.3 (5)
O5—V—O4—C15	−4.4 (3)	C7—N1—C8—C13	−178.1 (3)
O1—V—O4—C15	−106.9 (3)	V—N1—C8—C13	5.4 (3)
O2—V—O4—C15	94.8 (3)	C7—N1—C8—C9	1.7 (5)
N1—V—O4—C15	147.9 (4)	V—N1—C8—C9	−174.8 (3)
O3—V—O4—C15	174.4 (3)	C13—C8—C9—C10	−0.7 (5)
O5—V—N1—C7	−85.7 (3)	N1—C8—C9—C10	179.6 (3)
O4—V—N1—C7	121.4 (4)	C8—C9—C10—C11	0.2 (5)
O1—V—N1—C7	14.0 (3)	C8—C9—C10—Cl	−179.5 (2)
O2—V—N1—C7	176.2 (3)	C9—C10—C11—C12	0.0 (5)
O3—V—N1—C7	94.6 (3)	Cl—C10—C11—C12	179.7 (2)
O5—V—N1—C8	90.5 (2)	C10—C11—C12—C13	0.4 (5)
O4—V—N1—C8	−62.3 (4)	V—O2—C13—C12	171.7 (2)
O1—V—N1—C8	−169.7 (2)	V—O2—C13—C8	−8.8 (4)
O2—V—N1—C8	−7.54 (19)	C11—C12—C13—O2	178.6 (3)
O3—V—N1—C8	−89.1 (2)	C11—C12—C13—C8	−0.9 (5)
V—O1—C1—C6	21.3 (5)	C9—C8—C13—O2	−178.4 (3)
V—O1—C1—C2	−161.3 (2)	N1—C8—C13—O2	1.4 (4)
O1—C1—C2—C3	−176.2 (3)	C9—C8—C13—C12	1.0 (5)
C6—C1—C2—C3	1.3 (5)	N1—C8—C13—C12	−179.2 (3)
C1—C2—C3—C4	−0.8 (5)		

### Hydrogen-bond geometry (Å, °)

**Table:**

*D*—H···*A*	*D*—H	H···*A*	*D*···*A*	*D*—H···*A*
O3—H3···O2^i^	0.84 (1)	1.89 (2)	2.702 (3)	163 (5)

Symmetry codes: (i) −*x*+3/2, *y*−1/2, −*z*+3/2.
